# Exercise stress cardiovascular magnetic resonance imaging is feasible in adolescents and young adults with anomalous coronary arteries

**DOI:** 10.1016/j.jocmr.2025.101944

**Published:** 2025-08-20

**Authors:** Elizabeth L. Carter, Rebecca L. Moore, Kevin K. Whitehead, Sara L. Partington, David M. Biko, Danish Vaiyani, Mark A. Fogel, Matthew A. Harris, Julie A. Brothers

**Affiliations:** a Division of Cardiology, Department of Pediatrics, Children’s Hospital of Philadelphia, Philadelphia, Pennsylvania, USA; b Adult Congenital Heart Disease, Department of Cardiology, Hospital of the University of Pennsylvania, Philadelphia, Pennsylvania, USA; c Department of Radiology, Children’s Hospital of Philadelphia, Philadelphia, Pennsylvania, USA

**Keywords:** Exercise stress cardiac magnetic resonance imaging, Congenital heart disease, Coronary artery anomalies

## Abstract

**Aims:**

Anomalous aortic origin of a coronary artery (AAOCA) can result in sudden cardiac death in the young and risk stratification is challenging. Though dobutamine stress cardiovascular magnetic resonance (DS-CMR) is feasible in pediatric patients, exercise stress CMR (ES-CMR) may have lower rates of adverse events, higher diagnostic accuracy, and the ability to better reflect the physiologic changes occurring with exercise. We aimed to describe our institution’s experience with ES-CMR using supine bicycle ergometry in pediatric and young adult patients with AAOCA.

**Methods and Results:**

We retrospectively reviewed the medical records of AAOCA patients who underwent ES-CMR at our institution between 2011 and 2024 for demographic, clinical presentation, cardiopulmonary exercise test (CPET), and ES-CMR data. The exercise-based portion of the CMR consisted of supine cycle ergometry utilizing a ramp protocol, immediately after which ES perfusion imaging was performed. Fifteen minutes after stress imaging, rest perfusion imaging was acquired. Of 38 patients who underwent ES-CMR, the median age was 16 years (range 13–24) and 68% were male. Diagnoses included anomalous right coronary artery (n = 28), anomalous left coronary artery (n = 8), and single coronary artery (n = 1 single right, n = 1 single left). Median maximal heart rate (HR) during ES-CMR was 160 bpm (range 130–190, median 80% predicted) compared to a median maximal HR during patients’ most recent CPET of 187 bpm (range 160–203, median 97% predicted). No patients had perfusion defects at rest or with exercise stress, or evidence of myocardial scarring

**Conclusion:**

We demonstrate for the first time the use of ES-CMR in a cohort of pediatric and young adult patients with AAOCA. ES-CMR is a unique modality to assess for ischemia at rest and stress to assist with risk stratification by simulating physiologic changes with exercise stress. Although maximum heart rates during supine cycle ergometry are lower than those reached during CPET, they are similar to those reached during DS-CMR. ES-CMR is a valuable diagnostic tool and may be useful in the risk stratification of patients with AAOCA.

## Highlights

• **Key finding:** Exercise stress cardiac MRI is a feasible modality to diagnose and risk-stratify pediatric and young adults with anomalous coronary arteries.
• **Importance:** This is the first study to date describing the safety and feasibility of exercise stress cardiac MRI in the pediatric and young adult patient population.
• Exercise stress MRI is well tolerated with no side effects in our patient cohort.

## Graphical abstract

**Figure:**
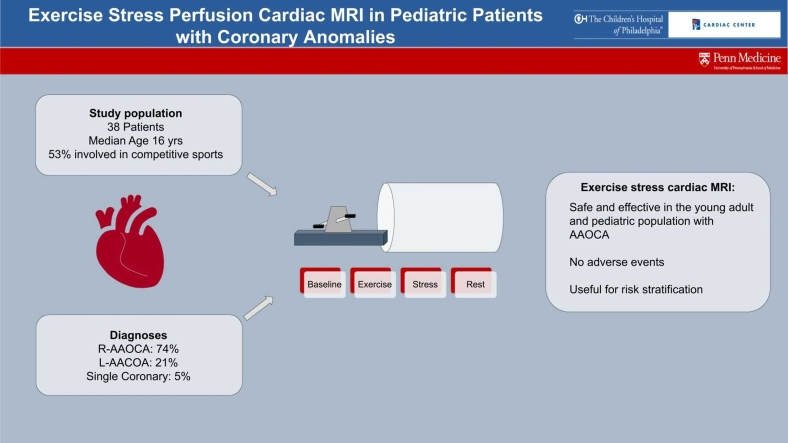
*R-AAOCA* Anomalous aortic origin of the right coronary artery, *L**-AAOCA* Anomalous aortic origin of the left coronary artery

## 1. Introduction

Coronary artery anomalies (CAA) include several different anatomic abnormalities in the origin, course, or termination of any of the main epicardial coronary arteries [1]. Anomalous aortic origin of a coronary artery (AAOCA) occurs when a coronary artery originates from or above the wrong aortic sinus. While many forms of AAOCA are of little hemodynamic or clinical consequence, there are subtypes that carry an increased risk of sudden cardiac arrest (SCA) or death (SCD), notably in young athletic individuals. Patients with AAOCA are commonly asymptomatic and are often diagnosed as an incidental finding on echocardiogram performed for benign murmur or family history of congenital heart disease; others may come to medical attention due to exertional symptoms or, rarely, SCA/D. While the exact mechanism of myocardial ischemia is incompletely elucidated, it is believed to be due to one or more of the following: coronary artery ostial stenosis or narrowing, oblique angle and/or high take-off from the aorta, length of intramural course, coronary artery spasm, and course behind the intercoronary pillar [2], [3]..

**Fig. 1:**
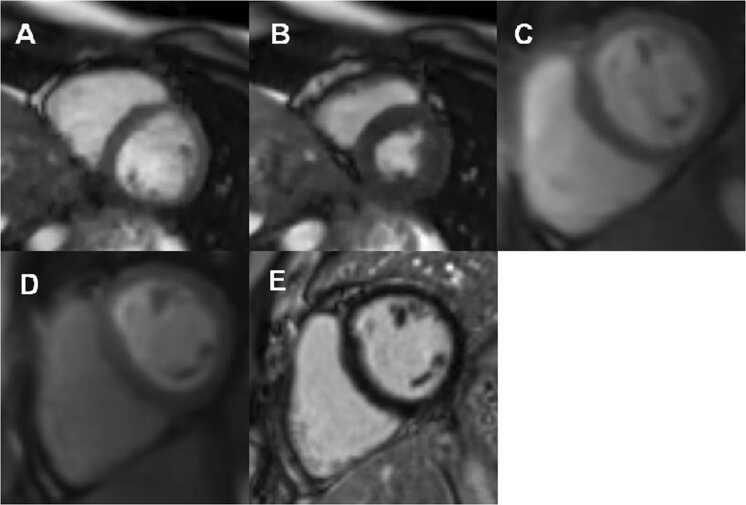
13- yo male with anomalous aortic origin of the right coronary artery with interarterial course. Mid short-axis view of the left ventricle. SSFP real-time cine images captured at end-diastole (A) and end systole (B) demonstrate dynamic LV shortening without wall motion abnormality (See also supplemental cine image for online use). First-pass gadolinium myocardial perfusion imaging acquired during exercise stress (C) and resting (D) conditions demonstrates normal homogenous myocardial perfusion without evidence of perfusion defects. Late gadolinium delayed enhancement imaging (E) demonstrates normal myocardial viability without evidence of myocardial scarring. *SSFP steady state free precession*

The management of asymptomatic patients with AAOCA, especially those with anomalous aortic origin of the right coronary artery (R-AAOCA), remains controversial, especially in cases where there are no obvious high risk features such as an intramural course, ostial narrowing or ostial stenosis. Because many patients with R-AAOCA are asymptomatic, it is incumbent on the cardiologist to further assess for SCA/D risk. Surgery is generally recommended in a patient who presents with SCA or who has signs and symptoms consistent with ischemia or complex ventricular arrhythmias; however, assessing ischemic risk in the asymptomatic young patient with R-AAOCA is challenging. On the other hand, most young patients with interarterial anomalous aortic origin of the left coronary artery (L-AAOCA) are referred for surgical repair regardless of symptoms. Other subtypes of L-AAOCA, including intraseptal course and coronary arising from the non-coronary sinus, are considered lower risk and are only referred for surgical repair with concerning cardiovascular symptoms or positive ischemia tests. Nearly a decade ago, recommendations were made regarding risk stratification in patients without evidence of ischemia or SCA which included stress testing with imaging, such as stress echocardiography or nuclear perfusion imaging, as part of the evaluation [2]. Over the past several years, stress cardiovascular magnetic resonance (CMR) has become more commonly used in the evaluation of patients with AAOCA, both in patients who do not undergo surgical repair as well as part of pre- and postoperative evaluation.

Recent studies have shown that dobutamine stress CMR (DS-CMR) is feasible in pediatric patients with AAOCA [4] and that impaired myocardial perfusion on pharmacologic stress CMR correlates with invasive fractional flow reserve (FFR) testing by cardiac catheterization [5]. Studies in adults have highlighted the advantages of exercise stress CMR (ES-CMR) given the higher rates of adverse events, contraindications, and potential side effects in pharmacologic stress protocols [6]. Exercise-based protocols may better mimic the hemodynamic and physiologic changes occurring with competitive sports participation, thus improving their diagnostic accuracy [7]. No studies to date have utilized ES-CMR in the young. Our aim is to describe our institution’s experience with exercise stress CMR in pediatric and young adult patients with AAOCA.

## 2. Methods

### 2.1. Study population and design

The study was determined to be exempt from review by our institutional review board. Informed consent was not required. A single‐center retrospective study was performed. Potential subjects were identified by query of our institution's radiology database for all patients less than 25 years old who underwent ES-CMR between January 1, 2011 and February 26, 2024. Patients were included if they had R-AAOCA, L-AAOCA or a single coronary artery. Patients were excluded if they had an alternative diagnosis that did not include anomalous origin of a coronary artery, or if there was complex congenital heart disease; patients with a history of isolated lesions such as partial anomalous pulmonary venous return (PAPVR), atrial or ventricular septal defects (ASD, VSD), patent ductus arteriosus (PDA) and non-critical aortic coarctation were included.

Baseline demographic data, including patient age, sex, race, height, weight, and BSA at the time of ES-CMR were collected from magnetic resonance imaging (MRI) reports. Patient diagnosis was confirmed and surgical history obtained by querying the electronic medical record. ES-CMR data were collected from existing reports. Cardiopulmonary exercise testing (CPET) data, including peak heart rate, presence of ectopy or ischemic changes on electrocardiogram (ECG), patient age, weight, height, and body surface area (BSA) at the time of study were collected from existing CPET reports. For patients with multiple CPET studies, the CPET performed closest in time to the ES-CMR was used. The percent predicted peak heart rate was calculated from normative exercise data published from our institution’s cardiopulmonary exercise laboratory [8].

### 2.2. ES-CMR protocol

The exercise-based portion of the CMR consisted of supine cycle ergometry utilizing a ramp protocol, which consists of a continuous ramped increase in work rate on the exercise bike until target heart rate is achieved (typically 0.85 × (220 bpm–age in years). Heart rate and oxygen saturation are monitored continuously. Blood pressure is measured at baseline and every 5 min while exercising and during recovery until the heart rate returns to baseline.

ES-CMR was performed on a 1.5T Siemens Magnetom scanner with an 18-channel phased array chest coil anteriorly and a 24-channel spine coil (Siemens Healthineers, Erlangen, Germany). Multiplanar steady-state free precession (SSFP) survey was acquired for determination of imaging planes. Then, baseline steady-state free precession cine imaging was performed in the short-axis view to assess ventricular volumes and ejection at rest, as well as phase-encoded velocity mapping across the aortic valve and main pulmonary artery. The patient remained in place to preserve localization and the CMR table was slid out of the scanning bore. Patients were then asked to exercise on the cycle ergometer attached to the table until their heart rate reached target after which the patient was promptly returned to the CMR scanning bore within approximately 10–15 s for real-time cine imaging, which was performed in the short axis and four-chamber views to assess for wall motion abnormalities. The CMR table was slid out of the scanning bore for a second time, and the patient was asked to resume cycling until the target heart rate was reached again, after which 0.08 cc/kg of gadobutrol contrast (Bayer AG, Leverkusen, Germany) was injected; the patient was slide back into the scanning bore and stress perfusion imaging was acquired. Stress perfusion imaging consisted of dynamic multi-phase SSFP sequence using generalized partially parallel autocalibrating acquisition (GRAPPA) and acceleration factor of two with motion correction. There was single average acquisition with trigger pulse of 1 and concatenations of 1 to 2 dependent on heart rate. The repetition time/echo time/flip angle were 3 ms/1 ms/50 degrees with a saturation delay of 110 ms. In-place resolution was 2 × 2 mm with a slice thickness of 8 mm. The perfusion sequence was acquired for 70 s. Typically, we acquire four slices, three in the short axis (base, mid, and apex) and a four-chamber view. Acquisition typically occurs with each heartbeat.

The patient was then allowed to rest for 15 min to allow their heart rate to return to baseline and 0.08 cc/kg of gadobutrol contrast was injected again for rest perfusion imaging. Finally, 0.04 cc/kg of gadobutrol contrast was injected and after 5 min, a SSFP inversion recovery late gadolinium enhancement (LGE) sequence was acquired for assessment of myocardial fibrosis.

## 3. Results

Of 38 patients who underwent ES-CMR, the median age at time of study was 16 years (range 13–24), and 68% were male. Diagnoses included R-AAOCA (n = 28, 73.6%), L-AAOCA (n = 8, 21.1%), and two patients with a single coronary artery origin (5.3%, 1 single off right sinus, 1 single off left sinus). Of the eight patients with L-AAOCA, four were anomalous left coronary artery arising from the right coronary sinus, two were left coronary artery arising from the non-coronary sinus and two were anomalous left coronary artery with intraseptal course. Two patients with L-AAOCA underwent surgical repair following the ES-CMR. Three patients with R-AAOCA had a history of surgical repair at the time of ES-CMR; one patient had right coronary artery unroofing and reattachment of the right-left commissure, one patient had right coronary artery unroofing, and one patient had repair of PAPVR but no intervention on the coronary arteries. The remainder of the patients (n = 35) had not undergone surgical intervention at the time of ES-CMR. Of the 38 total patients, 20 (52.6%) were involved in some level of competitive sports participation. Median maximal heart rate (HR) during ES-CMR was 160 bpm (range 130–190, median 80% predicted) compared to a median maximal HR during patients’ most recent CPET of 187 bpm (range 160–203, median 97% predicted). In patients with documented maximal HR data, 29% had max HRs >85% predicted value. No patients had perfusion defects at rest or with exercise stress, or evidence of myocardial scarring related to the coronary artery anomaly. Within our cohort, a full ES-CMR study took an average of 114 min. Two patients had symptoms at the time of ES-CMR; one patient felt “tired” while another complained of transient chest pain.

One patient with R-AAOCA and no history of intervention had an incidental finding of significant diffuse and mostly midmyocardial delayed enhancement along the lateral wall of the base of the left ventricle, extending to the mid ventricle (the delayed enhancement was not consistent with the right coronary artery territory) and a small amount of fluid overlying the delayed enhancement which suggested possible myocarditis. The etiology of this finding was unclear, but the patient had additional joint complaints suggesting a possible underlying rheumatologic disorder and the patient was managed as myocarditis. Additionally, there were some limitations to completing the ES-CMR; one patient’s height made positioning the supine bicycle challenging, leading to difficulty with gating and patient motion. Five studies were affected by patient movement or intolerance of remaining in the scanner. There were no adverse events reported. No patients had a documented serious cardiac event after ES-CMR on review of available medical record information at the time of last follow-up.

## 4. Discussion

Use of ES-CMR in congenital heart disease has been described previously in certain at-risk pediatric populations, including patients with repaired transposition of the great arteries or patients with Fontan physiology [6]. We demonstrate, for the first time, the use of ES-CMR in a cohort of pediatric and young adult patients with AAOCA, a population at risk for ischemic events. ES-CMR is a unique modality to assess for ischemia at rest and stress as a means of risk stratification and to simulate physiologic changes occurring with exercise stress in a single study. Expert consensus recommends exercise stress testing every one to three years in patients with AAOCA; ES-CMR could provide a useful adjunct to routine exercise testing in certain at-risk populations [2].

Pharmacologic stress testing is an alternative to ES-CMR. However, pharmacologic stress testing does not replicate the hemodynamic and neurohormonal changes that occur with physical exercise, which is preferred for stress imaging when feasible [6]. Additionally, the use of dobutamine or adenosine can be associated with adverse reactions such as hypotension or hypersensitivity. Vasodilators and adenosine are associated with bronchospasm due to alpha receptor activation and can be contraindicated in patients with reactive airway disease; additionally, patients with advanced conduction abnormalities or sinus node dysfunction should avoid these agents due to alpha agonist effects on heart rate and atrioventricular conduction [9]. Dobutamine has inotropic properties through stimulation of beta receptors and should be avoided in patients with hemodynamically significant LV outflow tract obstruction, certain atrial tachyarrhythmias, and recent myocardial infarction [9]. ES-CMR has fewer reported side effects, and at our institution, the ES-CMR procedure was well tolerated with no reported complications in our study cohort.

Despite the advantages of ES-CMR, it is not widely utilized due to the lack of commercially available CMR-compatible exercise equipment, cost, and the relative technical difficulty of ES-CMR compared to pharmacologic stress testing [6]. Additionally, prolonged scan times for ES-CMR may not be feasible at every center, and the length of scan may limit an individual center’s ability to readily utilize this diagnostic tool. Additionally, ES-CMR may have limitations with data acquisition and quality. Within our cohort, five studies were limited by motion artifact or patient intolerance; of those five, three of the patients had difficulty tolerating the study, which is not a problem unique to ES-CMR and can be seen in routine CMR studies as well. One patient’s height impacted positioning of the supine bicycle and made data acquisition difficult. However, patients with a similar height to this patient were able to complete the test without issues. Individual body habitus may impact success of the study in rare situations. Regardless of these concerns, all five studies with limitations were able to be completed with clinically interpretable imaging.

Although maximum heart rates during supine cycle ergometry were lower than those reached during CPET, the heart rates we describe were similar to the ranges reached during DS-CMR and the DS-CMR study was not designed to mimic peak exercise obtained during an exercise stress test [4]. Expert consensus recommends a maximal exercise stress test with additional imaging to help assess ischemic burden in patients with AAOCA after the diagnosis is made and we feel ES-CMR should not replace traditional exercise stress testing [2] but rather be a complementary testing modality to be utilized when possible. Its use may only be tenable for highly specialized pediatric centers, given prolonged scan times and necessary equipment and training.

## 5. Limitations

This single-center study is limited by the small sample size of patients and is subject to ascertainment bias. Further longitudinal studies should be performed with larger sample sizes and multiple institutions. Also, patients need to be of an age where they have the capability to complete the exercise testing in the CMR scanner, typically at least adolescence, and this limits the population able to undergo ES-CMR. Additionally, we examine a single time point for these patients who may have lifelong risk of adverse cardiac events and there should be further studies on this cohort of patients and the optimal way to risk stratify and monitor them over time. In general, stress imaging in patients with AAOCA has a low negative predictive value and study results are most helpful if they are positive [2]..

**Table 1:** Demographic information and most recent CPET testing results for the 38 included patients.

Study ID	Age at clinical presentation (y)	Sex	Race	Diagnosis	Surgical repair prior to CMR	Competitive athlete?	Nuclear perfusion scan	CPET date	Age at CPET (y)	Ectopy during CPET	ECG changes during CPET	CPET peak HR	CPET% predicted HR
1	9	M	White	Anomalous R coronary from left sinus with intramural course	RCA unroofing, reattachment of R-L commissure	N	Negative	February 7, 2008	11	None	None	171	
2	9	M	White	Anomalous R coronary from left sinus with intramural course	RCA unroofing, reattachment of R-L commissure	N	Negative	February 7, 2008	11	None	None	171	
3	16	F	Other	Single L coronary	None	Y	Negative	March 4, 2014	16	None	None		
4	Un-known	F	Asian	Anomalous R coronary from left sinus	None	N	None	September 9, 2010	9	None	None	203	96
5	16	M	White	Single R coronary	None	N	None	None					
6	16	M	White	Anomalous LMCA from the R sinus with prepulmonic LAD and retroaortic circumflex	None	Y	See note	July 21, 2020	17	None	None	179	94
7	15	M	Other	Anomalous LMCA from R sinus (RCA and LMCA from common trunk)	None	N	Negative	December 30, 2019	15	None	None	193	100
8	14	M	Black	Anomalous R coronary from the L sinus with likely intramural course	None	Y	Negative	July 24, 2020	19	None	None	181	98
9	4	M	White	Anomalous R coronary from the L sinus	None	Y	None	July 22, 2020	14	Single PVC	None	190	97
10	18	F	White	Anomalous R coronary from the L sinus with short intramural course	None	N	None	August 31, 2020	19	None	None	176	85
11	17	M	White	Anomalous L coronary from R sinus	None	N	Negative	January 2, 2019	21	None	None	173	94
12	19	M	Black	Anomalous L coronary from NC sinus	None	N	Negative	December 31, 2020	20	Single PAC	None	200	100
13	12	M	White	Anomalous R coronary from the L sinus with mild flattening of proximal RCA	None	N	None	August 6, 2021	19	None	None	160	85
14	12	M	White	Anomalous L coronary from R sinus with intraseptal/transeptal course	None	Y	Negative	July 21, 2021	16	None	None	184	90
15	15	M	White	Anomalous R coronary from the L sinus, possible intramural course	None	Y	None	November 17, 2021	18	See note	See note	187	100
16	16	F	White	Anomalous R coronary from the L sinus	None	Y	None	December 2, 2021	16	None	None	187	95
17	14	F	Asian	Anomalous R coronary from the L sinus with intramural course	None	Y	None	February 15, 2022	15	None	None	179	91
18	0	M	Other	Anomalous L coronary from R sinus	None	Y	None	January 18, 2022	13	None	None	200	100
19	17	M	White	Anomalous R coronary from the L sinus	None	N	Negative	June 20, 2022	24	None	None	193	100
20	4	M	White	Anomalous L coronary from R sinus (common trunk) with transeptal/intraseptal course	None	N	None	June 21, 2022	13	None	None	203	100
21	0	M	Black	Anomalous R coronary from the L sinus	None	N	None	February 7, 2022	16	PACs at rest	None	181	96
22	8	M	Black	Anomalous R coronary from the L sinus	PAPVR repair of LUPV to L innominate	N	Negative	June 28, 2022	17	None	None	187	100
23	13	F	Asian	Anomalous R coronary from the L sinus, possible intramural course, oblique angle at origin	None	Y	Negative	July 15, 2022	18	None	None	173	86
24	10	F	White	Anomalous R coronary from the L sinus	None	Y	Negative	None					
25	17	F	Asian	Anomalous R coronary from the L sinus	PDA device closure, aortic stent due to coarctation	N	None	July 19, 2022	19	Rare PVCs rest	ST depression in inferolateral leads	190	92
26	11	M	White	Anomalous R coronary from the L sinus, likely intramural course	None	Y	None	August 16, 2022	13	None	None	200	100
27	12	F	White	Anomalous R coronary from the L sinus, likely intramural course	None	Y	None	September 1, 2022	14	1 PAC 1 PVC	None	203	100
28	12	M	Other	Anomalous R coronary from the L sinus, possible intramural course, oblique angle at origin	None	Y	None	August 16, 2022	13	None	Limited by artifact	190	98
29	13	M	White	Anomalous R coronary from the L sinus	None	Y	Negative	December 8, 2022	18	None	None	190	100
30	5	M	White	Anomalous R coronary from the L sinus	None	Y	Negative	February 22, 2022	15	1 PVC at rest	None	196	100
31	16	M	Hispanic	Anomalous R coronary from the L sinus	coronary unroofing	Y	See note	January 27, 2021	17	1 PVC at rest 1 PVC at recovery	Anterolateral ST-segment depression	203	100
32	14	M	White	Anomalous R coronary from the L sinus	None	N	None	June 6, 2022	17	None	None	173	91
33	10	F	Black	Anomalous R coronary from the L sinus possible intramural course	None	N	Negative	April 26, 2023	14	None	None	187	97
34	13	M	Black	Anomalous R coronary from the L sinus	None	Y	Negative	May 16, 2023	13	None	None	184	96
35	6	M	White	Anomalous R coronary at junction of R/L sinus	None	Y	None	February 21, 2023	13	None	None	187	96
36	12	M	Other	Anomalous R coronary from the L sinus, likely short intramural course	None	N	None	August 4, 2023	16	Rare PVCs at rest	None	206	100

*CPET* cardiopulmonary exercise testing, *CMR* cardiovascular magnetic resonance imaging, *HR* heart rate, *PVC* premature ventricular contraction, *PAC* premature atrial contraction, *PAPVR* partial anomalous pulmonary venous return, *LUPV* left upper pulmonary vein, *NC* non-coronary

Notes: For patient 6, a nuclear stress test showed small mild reversible perfusion defect in mid anterior wall without wall motion abnormality, which was equivocal for ischemia and thought to be a false positive. For patient 15, there was significant motion artifact during CPET, could not exclude ectopy or ECG changes. For patient 31, a nuclear medicine scan showed a small region of mildly decreased myocardial perfusion in the inferior wall, which was partially reversible on resting imaging

**Table 2:** ES-CMR testing results and limitations, as well as long-term follow-up information regarding potential significant adverse events.

Study ID	CMR date	Age at CMR (y)	Max HR at exercise (bpm)	% Predicted of max HR	HR at CMR imaging (bpm)	Stress Perfusion defect	Stress Viability	Symptoms during scan	Length of scan (min)	Limitations	Date of last follow-up	Clinical outcome
1	June 15, 2011	14	150	77	130	Negative	Negative	No	74		2022	No documented events. Follow-up CMR negative.
2	August 7, 2013	16	150	78	100	Negative	Negative	No	87		2022	No documented events.
3	May 22, 2014	16	170	89	100	Negative	Negative	Tired	63		2025	Later diagnosed with POTS, had a loop recorder implanted from 2014−2016 with no events.
4	September 26, 2014	13	170	88		Negative	Negative	No	100			No documented events.
5	November 4, 2015	16				Negative	Negative	No	102		2019	No documented events.
6	November 26, 2019	16				Negative	Negative	No	121		2024	No documented events.
7	February 25, 2020	15				Negative	Negative	No	119		2025	Chest pain with coughing at follow-up.
8	July 24, 2020	19	160	80	120	Negative	Negative	No	114		2025	Follow-up CMR 2023 negative, had palpitations at follow-up, results of Holter pending.
9	July 22, 2020	14			163	Negative	Negative	No	90		2024	No documented events.
10	December 22, 2020	18			158	Negative	Negative	No	162		2025	Had an episode of syncope with pre-syncopal symptoms after standing for long time, thought to be vasovagal.
11	November 24, 2020	22	170	93	135	Negative	Negative	No	95		2025	No documented events.
12	November 19, 2020	20	170	92	110	Negative	Negative	No	159	Pt height made positioning the supine bicycle difficult	2023	Symptomatic during CPET, had negative troponin after CPET. Continued chest pain at rest and exercise. Underwent surgical repair with coronary unroofing in Mar 2021. Continued to have postoperative chest pain with negative troponin and ECG at follow-up.
13	August 6, 2021	19	140	80	100	Negative	Negative	No	122		2024	No documented events.
14	August 18, 2021	16	130	68	121	Negative	Negative	No	133		2024	No documented events.
15	December 3, 2021	18				Negative	See note.	No	158	Motion artifact	2024	No documented events.
16	December 2, 2021	16	160	80	130	Negative	Negative	No	170		2023	No documented events.
17	December 23, 2021	14	150	70	100	Negative	See note	No	145		2023	No documented events.
18	February 1, 2022	13	140	70	77	Negative	Negative	No	90		2022	Discussed surgical repair, further documentation unavailable.
19	February 21, 2022	24	172	96		Negative	See note	No	99		2023	No documented events.
20	April 26, 2022	13	160	80	120	Negative	Negative	No	76		2023	No documented events.
21	February 18, 2022	16				Negative	Negative	No	85	Uncooperative, high BSA	2023	No documented events.
22	June 28, 2022	17	145	71	102	Negative	Negative	No	123	Movement during CMR	2024	No documented events.
23	July 15, 2022	18				Negative	Negative	No	107		2022	No documented events.
24	July 22, 2022	15	165	88	140	Negative	Negative	No	106		2022	No documented events.
25	November 4, 2022	19	164	80		Negative	Negative	No	158		2025	Follow-up CPET in 2025 showed rare PVCs and 1 PAC, ST segment depression inferolateral leads that normalized by 1 min in recovery.
26	September 29, 2022	13	160	77		Negative	Negative	No	78		2025	No documented events.
27	September 1, 2022	14	190	96		Negative	Negative	No	114	Difficulty remaining in CMR	2024	Underwent coronary unroofing in November 2022, postoperative stress CMR with dobutamine in 2023 was negative.
28	November 3, 2022	14	170	80	110	Negative	Negative	No	78		2024	No documented events.
29	December 8, 2022	18	170	90	110	Negative	Negative	No	129		2021	No documented events.
30	December 29, 2022	15	180	94		Negative	Negative	No	119	Movement during CMR	2025	No documented events.
31	May 9, 2023	19	170	80	120	Negative	Negative	Transient chest pain, no ECG changes	96		2024	No documented events.
32	March 17, 2023	18	160	85	120	Negative	Negative	No	127	Frequent PVCs during CMR	2024	No documented events.
33	June 7, 2023	14	165	88		Negative	Negative	No	99		2024	No documented events.
34	July 28, 2023	13	138	72	90	Negative	Negative	No	118		2024	No documented events.
35	July 20, 2023	13	160	82	110	Negative	Negative	No	140		2024	No documented events.
36	August 4, 2023	16	130	68		Negative	Negative	No	101		2023	No documented events.

*CMR* cardiovascular magnetic resonance imaging, *HR* heart rate, *POTS* postural orthostatic tachycardia syndrome, *CPET* cardiopulmonary exercise testing, *BSA* body surface area, *PVC* premature ventricular contraction, *PAC* premature atrial contraction, *PDA* patent ductus arteriosus

Notes: For patient 15, there was mild basal anteroseptal hypokinesia at rest which was obliterated during exercise. For patient 17, a small amount of epicardial enhancement with possible slight subepicardial involvement was seen and could be consistent with prior myopericarditis. For patient 19, there was significant diffuse and mostly mid-myocardial delayed enhancement along the lateral wall of the LV base extending to the mid ventricle concerning for possible myocarditis

## 6. Conclusions

ES-CMR is a feasible modality for patients with AAOCA and we demonstrated no major adverse clinical events in a small patient population with negative ES-CMR testing. It avoids the potential side effects of pharmacologic stress testing and better reflects in vivo exercise conditions. For these reasons, ES-CMR may serve as a helpful adjunctive modality to assess for myocardial ischemia with exercise in this at-risk population.

## Funding

None.

## Author contributions

**Elizabeth L. Carter:** Writing – review & editing, writing – original draft, visualization, validation, investigation, formal analysis, data curation, conceptualization. **Rebecca L. Moore:** Writing – original draft, validation, resources, investigation, formal analysis, data curation, conceptualization. **Kevin K. Whitehead:** Writing – review & editing, validation, supervision. **Sara L. Partington:** Writing – review & editing, validation, supervision. **David M. Biko:** Writing – review & editing, visualization, supervision. **Danish Vaiyani:** Writing – review & editing, supervision, resources. **Mark A. Fogel:** Writing – review & editing, supervision. **Matthew A. Harris:** Writing – review & editing, validation, supervision, resources. **Julie A. Brothers:** Writing – review & editing, writing – original draft, visualization, validation, supervision, formal analysis, conceptualization.

## Declaration of competing interests

The authors declare that they have no known competing financial interests or personal relationships that could have appeared to influence the work reported in this paper.
